# Short- and long-term experience in pulmonary vein segmental ostial ablation for paroxysmal atrial fibrillation*

**Published:** 2006-01-01

**Authors:** H Pürerfellner, J Aichinger, M Martinek, HJ Nesser, J Janssen

**Affiliations:** Public Hospital Elisabethinen, Academic Teaching Hospital, Cardiological Department, Linz, Austria; † Medtronic Inc. , Bakken Research Center, Maastricht, Holland

**Keywords:** atrial fibrillation, pulmonary vein ablation

## Abstract

**Introduction:**

Segmental ostial pulmonary vein isolation (PVI) is considered a potentially curative therapeutic approach in the treatment of paroxysmal atrial fibrillation (PAF). There is only limited data available on the long-term effect of this procedure.

**Methods:**

Patients (Pts) underwent a regular clinical follow up visit at 3, 6 and 24 months after PVI. Clinical success was classified as complete (i.e. no arrhythmia recurrences, no antiarrhythmic drug), partial (i.e. no/only few recurrences, on drug) or as a failure (no benefit). The clinical responder rate (CRR) was determined by combining complete and partial success.

**Results:**

117 patients (96 male, 21 female), aged 51±11 years (range 25 to 73) underwent a total of 166 procedures (1.4/patient) in 2-4 pulmonary veins (PV). 115 patients (98%) had AF, 2 patients presented with regular PV atrial tachycardia. ,109/115 patients. exhibited PAF as the primary arrhythmia (versus persistent AF). A total of 113 patients with PVI in the years 2001 to 2003 were evaluated for their CRR after 6 (3) months. A single intervention was carried out in 63 patients (55.8%), two interventions were performed in 45 patients (39.8%) and three interventions in 5 patients (4.4%). The clinical response demonstrated a complete success of 52% (59 patients), a partial success of 26% (29 patients) and a failure rate of 22% (25 patients), leading to a CRR of 78% (88 patients). Ostial PVI in all 4 PVs exhibited a tendency towards higher curative success rates (54% versus 44% in patients with 3 PVs ablated for the 6 month follow up). Long-term clinical outcome was evaluated in 39 patients with an ablation attempt at 3 PVs only (excluding the right inferior PV in our early experience) and a mean clinical follow up of 21±6 months. At this point in time the success rate was 41% (complete, 16 patients) and 21% (partial, 8 patients), respectively, adding up to a CRR of 62% (24 patients). In total, 20 patients (17.1%) had either a single or 2 (3 patients, 2.6%) complications independent of the number of procedures performed with PV stenosis as the leading cause (7.7%).

**Conclusion:**

The CRR of patients with medical refractory PAF in our patient cohort is 78% at the 6 month follow up. PV stenosis is the main cause for procedure-related complications. Ablation of all 4 PV exhibits a tendency towards higher complete success rates despite equal CRR. Calculation of the clinical response after a mid- to long-term follow of 21±6 months in those patients with an ostial PVI in only 3 pulmonary veins (sparing the right inferior PV) shows a further reduction to 62%, exclusively caused by a drop in patients with a former partial success. To evaluate the long-term clinical benefit of segmental ostial PVI in comparison with other ablation techniques, more extended follow up periods are mandatory, including a larger study cohort and a detailed description of procedural parameters.

## Introduction

Based on groundbreaking work by Michel Haissaguerre et al. a catheter-based procedure was recently introduced into clinical routine to treat drug-refractory paroxysmal atrial fibrillation (PAF) [1,2]. During segmental ostial pulmonary vein isolation (PVI), electrically conducting myocardial extensions bridging the pulmonary veins (PV) with the posterior wall of the left atrium (LA) are disconnected to prevent the initiation (and the maintenance) of PAF.

Success rates reported in recent literature differ to a big extent (between 50 and 90%) [3,4] and are not always comparable, as applied techniques may vary considerably and are changing with growing experience in the same group. Moreover, evaluation of success is not consistent within published reports, as clinical judgement of ablation outcome may be difficult to determine (missing ECG documentation in symptomatic periods, asymptomatic episodes). In addition, follow up periods are rarely comparable and sometimes are rather short, so that there is still a lack of data addressing the long-term outcome in ablation of the PV ostia to treat PAF nowadays.

We have reported on our early experience in the past [5]. In this paper we report on our own short and long-term results with special consideration of distinct procedural parameters which have been developed in a time period of three years from 2001 to 2004.

## Patients and Methods

### Lasso Procedure

Briefly, after puncture of one (or both) femoral and the left subclavian (in case the coronary sinus can not be entered by a femoral access) vein(s) a multipolar electrode catheter is introduced in the coronary sinus to record left atrial activity. Thereafter, a (usually double) transseptal puncture is performed followed by pulmonary venography. Next, two electrode catheters are placed in the LA via two long sheaths, one for ablation (Celsius THERMOCOOL 7F, Biosense Webster, Inc.) and one circular decapolar catheter (“Lasso”, Biosense Webster, Inc.) to record electrical activity of myocardial extensions connecting the LA with a PV. The lasso catheter is situated as proximal as possible inside the PV whereas the ablations catheter is positioned at the very os to segmentally isolate the PV, thereby preventing stenosis which may occur when delivering energy deep inside a PV. Electrical entrance block from the LA to the PV is considered as the endpoint of the procedure which is documented by the elimination of PV potentials on the Lasso catheter. We have been using irrigated ablation catheters from the very beginning of our PV ablation experience, delivering up to 30 Watts of energy in the superior PVs (left superior, LSPV and right superior, RSPV) and a maximum of 20-25 Watts in the inferior PVs (left inferior, LIPV and right inferior, RIPV). The approach of targeting a PV for ablation is “empiric” i.e. independent of its arrhythmic activity during the time of the procedure. In the time period from 2001 to the first half of 2002, 3 PVs were routinely isolated, whereas since then all 4 PVs (including the RIPV which is sometimes more difficult to reach) were targeted during a single procedure.

### Follow up

In the following we report on our experience in ostial PVI in the time period between 12/2000 and 02/2004. The last follow up visit was performed in 04/2004. After the procedure, patients were monitored on an outpatient basis after one month and on an inpatient basis after 3, 6 and approximately 24 months. The following tests were routinely performed: Clinical examination, Holter-ECG, evaluation of quality of life using a standardized questionnaire, in addition (on an inpatient basis) transthoracic and transesophageal echocardiography, stress test, spiral computed tomography (CT) of the PVs, and (only in case of significant PV stenosis) magnetic resonance imaging of the PV and a lung scan. 

### Success criteria

Two distinct time periods were analyzed in order to evaluate the clinical outcome: a short term follow up period of 6 months (3 months for the last patients in this series) and a long-term follow up of approximately 24 months. The clinical outcome was classified as complete success (CS) if patients were without any documented arrhythmias and free of antiarrythmic medication. A partial success (PS) was defined by an absence of clinical symptoms while patients being still on an antiarrhythmic drug. These two groups comprised the patient cohort for which the clinical response rate (CRR) was calculated. A clinical failure (CF) was classified as such in the rest of patients who showed no benefit while usually still on drug.

## Results

### Demographics

117 patients (96 male, 21 female) aged 51±11 years (range 25-73 years) were included for chronic follow up. 26 patients (22%) had an underlying heart disease (hypertension n=18, coronary artery disease n=4, diabetes n=4); 2 patients had a history of a transient ischemic cerebral attack. Patients had used a mean number of 3.1±1.4 antiarrhythmic drugs. In most instances several class I, II and class III drugs had been administered previously (propafenone 70%, betablocker 65%, amiodarone 56%, sotalol 51%, flecainide 26%); uncommonly, class IV drugs (verapamil in 18% and digoxin in 14%).

### Arrhythmia characteristics

Arrhythmia history showed a wide inter-individual variation and lasted for 73±67 months (median 48 months), the maximal episode duration within the last 3 months had a median of 390 min, the mean number of arrhythmia recurrences acccounted for n=2/week. Table 1 illustrates the classification of the preexisting arrhythmia as defined by the surface ECG. 115/117 patients (98%) exhibited AF as their primary clinical atrial tachyarrhythmia with 86 presenting only with PAF, 10 showing additional regularized atrial tachycardias, 14 typical atrial flutter and 5 patients presenting both types, respectively. The majority of patients (109/115, 94.7%) presented with PAF, 6/115 (5.3%) showed only persisting AF . Two patients (1.6%) exhibited nearly incessant “P on T” atrial runs suggesting a pulmonary venous origin which was reconfirmed during the ablation procedure. These 2 patients underwent pulmonary venous segmental ostial ablation and therefore were both included in this analysis.

### Procedures

166 procedures (1.42/patient) in 2-4 PVs were performed in total, consisting of 117 (71%) primary, 44 (27%) secondary und 5 (2%) tertiary procedures. Figure 1 illustrates the respective numbers of primary, secondary and tertiary procedures per patient and year (of first procedure) between 2001 and 2003. In 63 patients (55.8%) a single procedure was carried out, as opposed to 45 patients (39.8%) with a second and 5 patients (4.4%) with a third ablation procedure. The duration of the primary procedure lasted for 228±55 min and was significantly longer in comparison with the secondary (206±56 min, p=0.004). The fluoroscopic time duration for the primary and secondary procedure accounted for 62±15min, and 53±20min, respectively.

In most of the patients the LSPV (96%), LIPV (96%) and the RSPV (95%) were isolated ostially, whereas the RIPV was targeted in only 55%. The reason for this is that this PV is usually more difficult to reach and was spared in our early ablation experience from 2001 to the first half of the year 2002. Consequently, the number of patients with PVI in 3PVs, 4 PVs or 2 PVs accounted for 40 % (n=47), 54% (n=63) and 6% (n=7), respectively.

### Success rates

113 patients were evaluated for the determination of the clinical outcome after 6 months (after 3 months only in 8/113 patients with a limited follow up). CRR accounted for 78% (88 patients) consisting of 52% CS (59 patients), 26% PS (29 patients) und 22% CF (25 patients), respectively (Figure 2). CS was higher in patients undergoing PVI in 4 PVs versus PVI in 3 PVs (54% versus 44%) (Figure 3). However, the CRR was similar in both groups (77% in 4 PVs and 78% in 3 PVs, respectively).

For the determination of the long term succes rate after a mean of 21±6 months only those patients with PVI in 3 PVs (sparing the RIPV) were evaluated. For a total of 39 patients the CS, PS and CF rates accounted for 41% (n=16), 21% (n=8), 38% (n=15), respectively, leading to a CRR of 62% (n=24). Considering only this group of 39 patients for their specific clinical course after 6 months separately, CS, PS and CF rates account for 41% (n=16), 33% (n=13) and 26% (n=10), respectively, leading to a CRR of 74% (n=29) (Figure 2). From these results one may conclude that CRR in the longterm follow up is reduced by a reduction in PS, however, the CS rate seems stable even in the long run.

The clinical success rates per year of experience are depicted in Figure 4. As patients had to have all procedures performed within a single year for this analysis, the number of patients evaluated decreases to n=103.

### Complications

Significant PV stenoses are the leading cause of side effects in the longterm follow up ranging up to 7.7% (n=9), all other complications account for less than 1.5%, such as pericardial tamponade (n=2), pericardial effusion (n=2), stroke (n=2), pneumo-/hemothorax (n=2), groin hematoma (n=2) and pericarditis (n=1). In total, 20 patients (17.1%) had either a single or 2 (3 patients, 2.6%) complications independent of the number of procedures performed.

## Discussion

We have reported repeatedly on our experience in ostial PVI recently, in particular on the occurence of PV stenosis [6,7], quality of life [8] and the evaluation of procedural success by use of longterm implantable monitoring provided by special implantable pacemakers with extended storage capabilities in chronically paced patients [9].

In this paper our data highlight the potentially curative therapeutic approach of segmental ostial PVI over a short- and longterm follow up in a patient cohort with highly symptomatic and medically drug-refractory recurrent PAF and without significant underlying heart disease. In total, 78% of patients show a clinical benefit over a follow up period of 6 months, which is reduced to 62% in the long-term after approximately 24 months in a patient cohort that underwent PVI in 3 PVs (sparing the RIPV). An ablation procedure that includes PVI in the RIPV as well exhibits a higher curative success rate after 6 months of follow up (54% versus 44%).

### Segmental ostial PVI

According to current knowledge, a segmental ostial ablation approach at the PV-LA junction represents a predominant trigger elimination of ectopic foci from within the PVs capable of initiating PAF by rapid focal discharges. To which extent an ongoing fibrillatory process in the LA may be maintained by such PV discharges remains an open issue at this point in time [10]. In addition, ectopic activity that may trigger PAF may also be generated at different sites in both atria (posterior and anterior LA, coronary sinus, terminal crest, superior caval vein). This may well explain why a 100% cure rate is unlikely when applying radiofrequency ablation at the ostial level of the PVs solely.

### Procedural parameters

Despite the fact that the arrhythmogenic potential of the RIPV was somewhat in doubt years ago, our results are in accordance with other more recent reports in the literature demonstrating the importance of implementing this PV in the ablation procedure. As illustrated in Figure 3 the CS is higher when isolating all 4 PVs (versus 3 PVs). The RIPV is sometimes more difficult to reach technically, however, with improved catheter steerability this problems is solved most of the time nowadays. Other procedural parameters do not seem crucial in our series as our approach of isolating the PVs did not change over the years using the same diagnostic catheter (Lasso catheter) within the PV ostium and a stable energy source for ablation (radiofrequency ablation with irrigated catheters) with unchanged energy settings (30 Watts for the superior PVs and 20-25 Watts for the inferior PVs).

### Longterm success rate

Our longterm success rates after approximately 24 months may be limited by the fact that these results represent the outcome in patients having undergone ostial PVI in 3 PVs exclusively. If we compare the longterm success rate of this separted group with its 6 month follow up data, it is obvious that the CS is literally idenitical (41%) whereas the PS and the CRR seem to be somewhat higher on a short term basis (33% versus 21% and 74% versus 62%, respectively). Our results demonstrating a stable cure rate over an extendend period are confirmed in a previously published larger report [11]. However, it remains interesting to reconfirm those data by our own series of patients having undergone a 4 PV ablation approach from the second half ot the year 2002 onwards.

### Learning curve

To find an appropriate measure for our learning curve we have analyzed the number of secondary procedures and the success rates . As depicted in Figure 1 there is a constant decrease of secondary procedures per patient with growing experience: The percentage decreases from about 50% in patients with their primary procedure in the year 2001 to 40% and 25%, respectively in patients who underwent PVI in the year 2002 and 2003. The reason for this is based on the growing knowledge about the nature of early recurrences (within the first days after the primary procedure): As those recurrences may merely represent transient effects of leason healing we tended to treat those arrhythmias conservatively with growing experience by administering antiarrhythmic drugs instead of performing an early redo procedure. In addition , we were able to exactly quantify and publish the decrease of arrhythmic burden of PAF within the first 3 months after PVI in 12 patients who were previously implanted with a last generation implantable pacemaker offering extended storage capabilities (Medtronic Inc, AT 500) [9]. Figure 4 demonstrates the success rate based on the year of the primary procedure. It shows an increase in the CS from 40% in the year 2001 to about 50% in the next two years. The most likely cause for this is the fact that the procedure changed from a 3 PV to a 4 PV isolation approach (including the RIPV) . The CRR, however, remains fairly constant, reaching 70 to 80%.

### Redo procedures

In general, concomitant atrial tachycardias or atrial flutter were not a primary target for ablation during the first ablation session. In case of a recurrence of PAF, one or more PVs were usually reconnected and had to be reisolated. In addition, sustained atrial tachycardias as well as right or left atrial flutter circuits were mapped as appropriate. However, no detailed ablation results for these substrates are available in this reported patient series.

### Complications

With regard to diagnosis, management and outcome in PV stenosis follwing ostial PVI we may refer to our previously published results [6,7]. Despite the fact that there may be multiple factors operational for the occurence of PV stenosis (energy source, amount of delivered energy, localisation of the affected PV) the exact site of energy delivery at the ostial level seems to be of highest importance: The more energy delivered within a PV (versus ostial) the higher the chance of developing significant PV stenosis. In general, the symptom threshold (including dyspnea, hemoptysis and pneumonia) for significant PV narrowing exceeds 60% of luminal narrowing, however, not every single significant PV narrowing produces symptoms. Lung scanning is a useful diagnostic test to document hemodynamically significant PV stenosis by detecting a segmental perfusion deficit. Interventions in significant PV stenosis including PV dilatation and/or stenting may lead to a high reocclusion rate. In our published series of 6 patients with predominant single PV stenosis the clinical outcome is beneficial despite reocclusion in 2 patients during a longterm follow up.

### Limitations

Longterm success is based on the results in patients with a 3 PV ostial isolation approach (sparing the RIPV). It may well be that chronic results in patients with a 4 PV (including the RIPV) approach are superior.

Although routine longterm ECG monitoring was performed at prespecified points in time during follow up, clinical success is primarily based on symptomatic episodes of PAF. It remains unclear who often and to what extent silent episodes of PAF may have changed the clinical results of PVI in our patient subgroups.

### Future outlook

According to many other working groups worldwide we have changed our ablation strategy recently: Using a 3 dimensional mapping system (CARTO, Biosense Webster Inc.) an electroanatomic map of the LA including the mitral valve annulus and the PVs is reconstructed pre ablation. Thereafter, periostial circumferential atrial lesions are deployed around the septal and lateral PVs in order to modify the substrate of PAF (Figure 5). In addition, an ablation line in between the LIPV and the mitral valve is drawn (so called "mitral isthmus"), which is at times accompanied by a roof line in the LA connecting the superior PVs. It seems critical to attain complete lesion sets designed to prevent the spread of focal discharges from withhin the PV to the rest of the LA (Pappone approach [12]). At this point in time we are still using a multipolar circular catheter to be located at the ostial level to monitor and guide PV isolation at the LA-PV junction. Recently published reports demonstrate higher success rates using circumferential PV atrial ablation in contrast to segmental ostial PVI both in PAF [13] and (even more) in persistent to permanent forms of AF. An additional advantage of this technique is based on the diminished occurence of PV stenoses by applying energy >1cm away from the ostial level. However, additional point lesions at the ostial level of a PV are often required to accomplish complete LA-PV block.

In the meantime a multislice CT imaging modality is available in our institution which enables the 3 dimensional representation of a patient’s individual cardiac anatomy (Figure 6). In addition, worldwide intensive collaboration within different manufacturers of cardiac visualization systems is ongoing to enable "3 D image integration" which will allow a direct transfer of imaging files to the mapping system at work. By this, a direct transfer of the individual electrical activation to the individual patient’s anatomy seems feasible ("image guiding").

Moreover, interesting and promising efforts are made by one manufacturer (Stereotaxis, Inc) to conduct an ablation procedure by remote navigation of the ablation catheter which is directed by a strong magnetic field around the patient’s body.

## Conclusion

The CRR in patients with medically refractory PAF undergoing ostial PVI accounts for 78% after a short term follow up of 6 months. A 4 PV procedure (including the RIPV) warrants a higher CS. In patients with a 3 PV procedure (sparing the RIPV) the CRR in the longterm follow up is reduced to 62%. However, the CS rate seems to be constant, whereas the PS is reduced. PV stenosis is the main cause for complications. In addition to an exact analysis of procedural parameters a longer follow up period seems necessary to better quantify the clinical benefit to risk ratio of segmental ostial PVI in contrast to other strategies in the treatment of atrial fibrillation.

## Figures and Tables

**Table 1:**
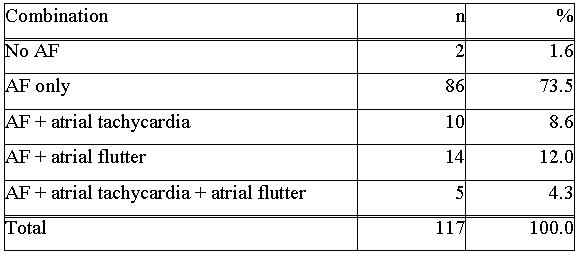
Classification of the preexisting arrhythmia (AF=atrial fibrillation)

**Figure 1:**
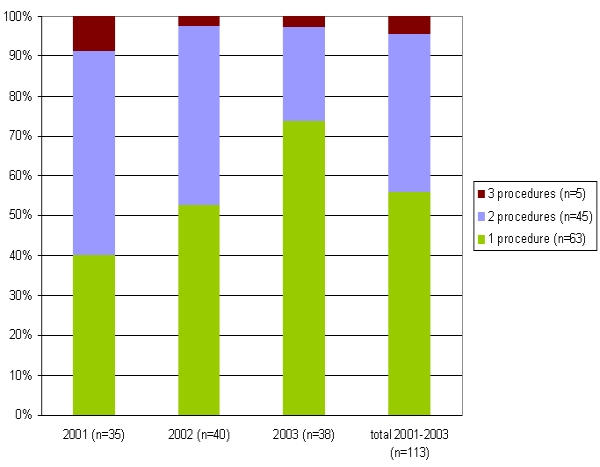
Relative percentage of primary, scondary and teriary procedures per patient and per year of ablation (of the primary procedure)

**Figure 2:**
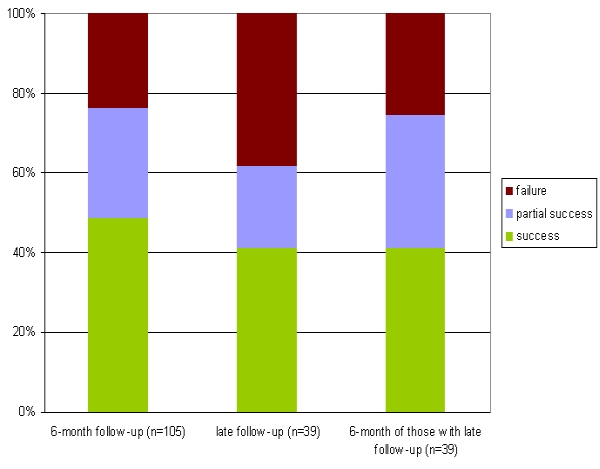
Success rates in the short- and longterm follow up period

**Figure 3:**
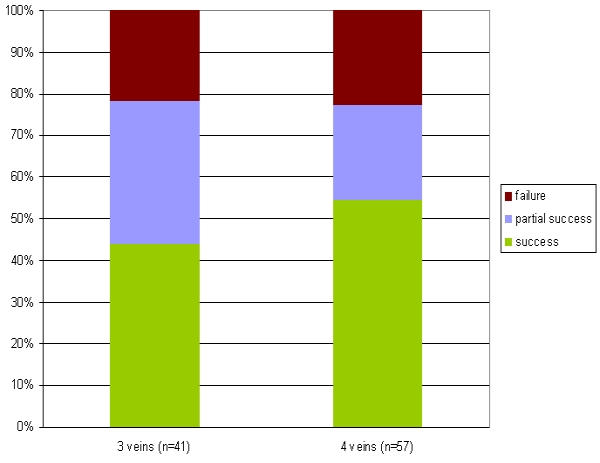
Success rates in a 3 versus a 4 PV approach (including the RIPV)

**Figure 4:**
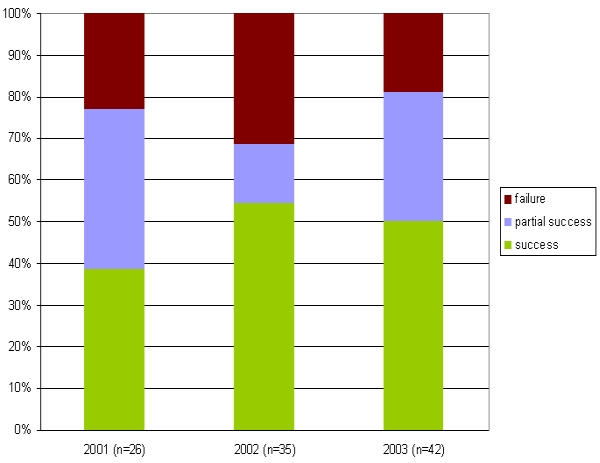
Success rates per year of ablation performed

**a:**
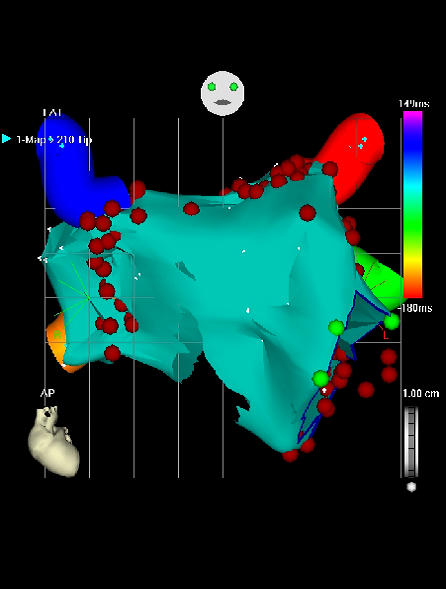


**b:**
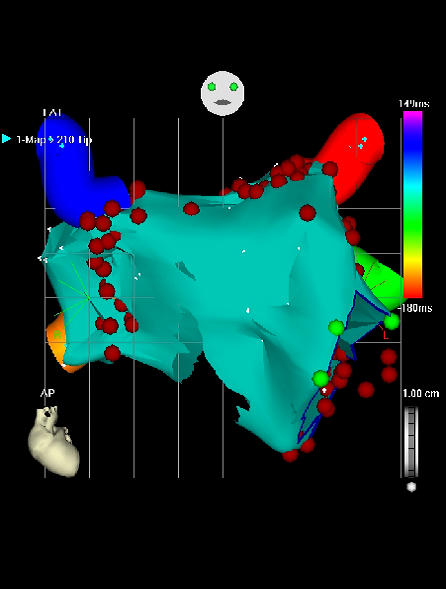


**Figure 6:**
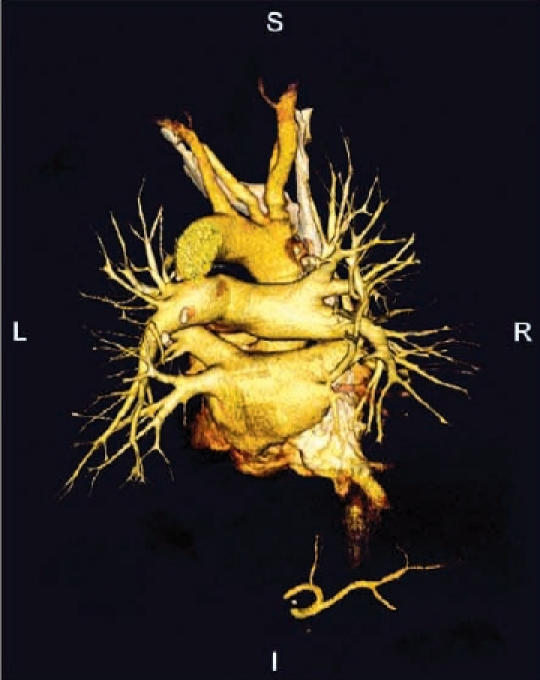
Multislice CT demonstrating aortic (above), pulmonary arterial (mid) and pulmonary venous vasculature (posterioanterior projection)
